# The association of nonalcoholic fatty liver disease with bone mineral density in type 2 diabetes

**DOI:** 10.1186/s40001-022-00775-z

**Published:** 2022-08-08

**Authors:** Juan Du, Yan Ma, Hongmei Lang, Changquan Huang, Xingping Zhang

**Affiliations:** 1 Department of General Medicine, Chengdu Second People’s Hospital, Chengdu, 610000 China; 2 Department of Geriatrics, Chengdu Second People’s Hospital Chengdu, Chengdu, 610000 China

**Keywords:** Nonalcoholic fatty liver, Diabetes, Lumbar, Bone mineral density

## Abstract

**Objective:**

We examined the association between nonalcoholic fatty liver disease and lumbar spine bone mineral density in individuals with and without type 2 diabetes.

**Methods:**

The lumbar BMD of 1088 subjects was measured using dual-energy X-ray absorptiometry (DXA). Liver fat content was quantified via B-mode ultrasound. Multivariable linear regression was used to study the association between NAFLD and lumbar BMD in participants with and without T2DM.

**Results:**

The lumbar BMD in the T2DM group and the non-diabetes group was higher in the NAFLD group than in the non-NAFLD group (*P* < 0.001). Multivariate regression analysis in the T2DM group showed that after adjusting for confounders, the positive association between lumbar spine BMD and NAFLD remained (*P* = 0.027). In the non-diabetes group, after adjusting for confounders, the association between NAFLD and lumbar spine BMD disappeared.

**Conclusions:**

The relationship between nonalcoholic fatty liver disease and lumbar bone mineral density may differ in individuals with and without diabetes. The effect of nonalcoholic fatty liver disease on bone mineral density needs to be evaluated in different clinical contexts.

## Introduction

Nonalcoholic fatty liver (NAFLD) is a disease in which excessive fat deposits in liver cells in the absence of excessive drinking or other causes of liver damage and is related to hepatic lipotoxicity [1]. Nonalcoholic fatty liver disease is not only associated with liver diseases such as nonalcoholic steatohepatitis, liver cirrhosis, and liver cancer, but also with increased prevalence of metabolic diseases such as hypertension, dyslipidemia, obesity, and T2DM (type 2 diabetes) [2–4]. Insulin resistance and obesity are the key pathogenic factors for nonalcoholic fatty liver disease and type 2 diabetes [5, 6]. Therefore, these two diseases usually coexist. Studies have indicated that 75% of type 2 diabetes patients have nonalcoholic fatty liver disease [7, 8]. The liver and bones are both active endocrine organs that have various metabolic functions [9]. Currently, clear evidence suggests that the bone mineral density of type 2 diabetes patients is higher than that of non-diabetes people, especially in the spine and hips. However, of type 2 diabetes is associated with an increased risk of fractures [10]. Some studies have suggested that there is a latent association between nonalcoholic fatty liver disease and bone mineral density. In addition to osteoporosis, which is commonly thought of as an age-dependent disease, other latent factors are associated with liver and bone tissue [11].

Although some previous studies have separately examined the effects of nonalcoholic fatty liver disease and diabetes on bone mineral density, there is little work discussing the impact of nonalcoholic fatty liver disease coexisting with type 2 diabetes on bone mineral density. Moreover, most of the previous studies concentrated in specific groups, such as postmenopausal women and obese adolescents. Therefore, in this study, we examined the association between bone mineral density and nonalcoholic fatty liver disease in type 2 diabetes.

## Subjects and methods

### Ethics statement

The study protocol was approved by the Ethics Committee of Chengdu Second People's Hospital (No: 2022144) and the requirement for informed consent was waived because of the retrospective nature of the study. All procedures performed in studies involving human participants were in accordance with the ethical standards of the institutional research committee and with the Helsinki declaration and its later amendments or comparable ethical standards.

### Subjects

This study included 1300 subjects who underwent dual-energy X-ray absorptiometry (DXA) and abdominal ultrasonography between 2016 and 2021. The T2DM subjects’ group was from the endocrinology department of our hospital, and the non-diabetes group was from the physical examination center of our hospital, which was matched with the age and sex of the T2DM group. The results of the first laboratory examination on admission in the T2DM group were selected, and the interval between BMD and ultrasound examination did not exceed one week. In the non-diabetes group, all the examinations were completed on the same day.

#### Inclusion criteria

(1) All participants were ≥ 18 years old; (2) nonalcoholic fatty liver disease patients were diagnosed with an ultrasound examination; (3) BMD was measured by dual-energy X-ray absorptiometry.

#### Exclusion criteria

(1) Autoimmune, viral or drug-induced hepatitis disease; (2) excessive alcohol consumption (over 20 g per day); (3) patients with diabetes other than T2DM; (4) patients with other diseases (hyperthyroidism, hyperparathyroidism, malignant tumors, etc.) that may affect BMD; (5) long-term use of drugs that affect BMD (such as glucocorticoids, steroids).

**Fig. 1:**
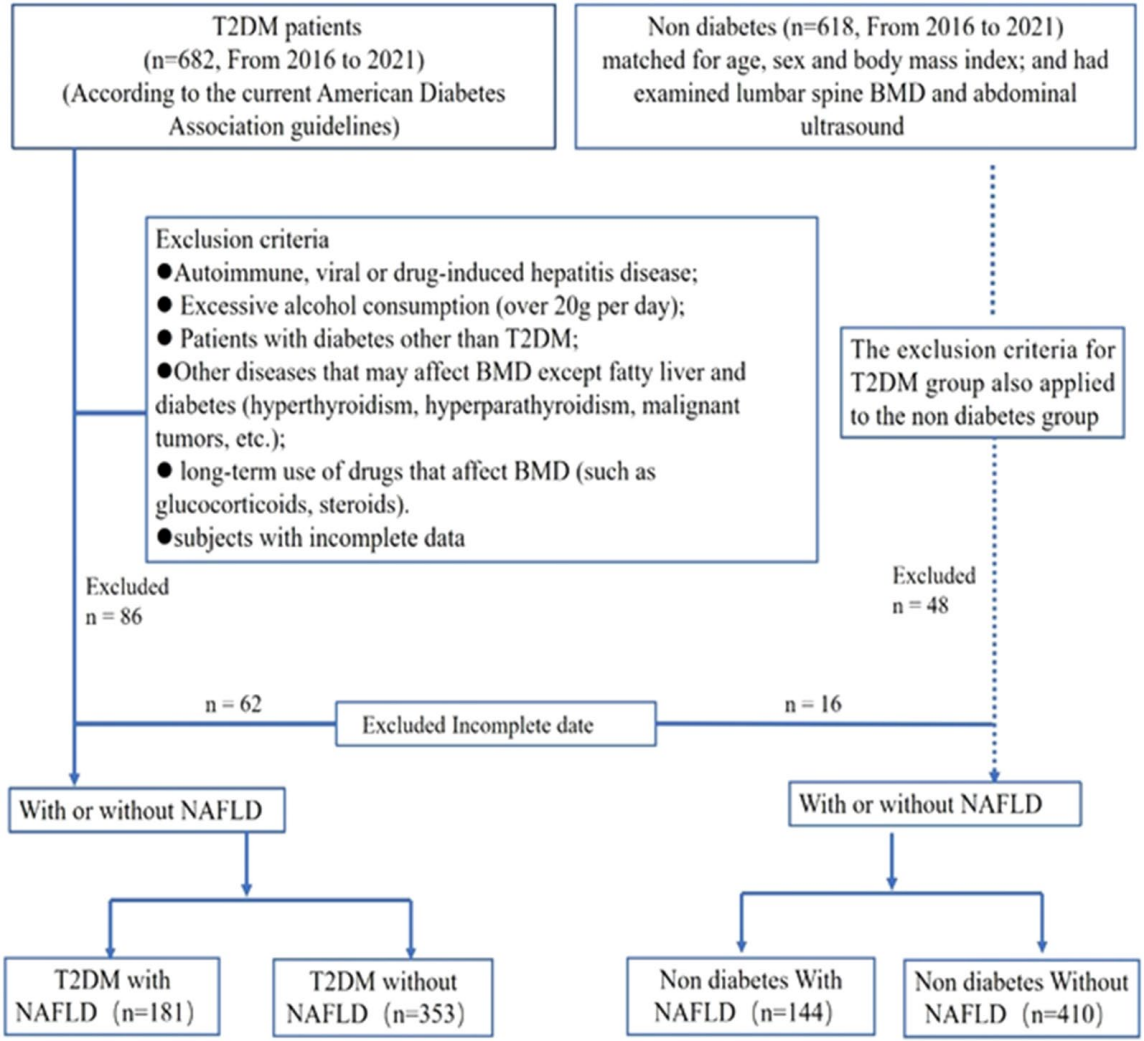
Flow diagram of study enrollment

After excluding subjects who did not meet the criteria or we had incomplete data, 1088 subjects were included. The subjects were divided into the T2DM group and the non-diabetes group. The diagnosis of T2DM was based on the recommendations of the current guidelines of the American Diabetes Association [12]. The patients in T2DM group were divided into a T2DM with NAFLD group (181subjects) and a T2DM without NAFLD group (353 subjects). The non-diabetes group was divided into the NAFLD group (144 subjects) and the non-NAFLD group (410 subjects). The subject selection and inclusion process is shown in Fig. 1.

### Dual-energy X-ray absorptiometry to measure lumbar spine BMD

According to the World Health Organization (WHO) diagnostic criteria, the T-score, Z-score and BMD value at the lumbar spine (L1–L4) were measured using DXA (GE Lunar Health Care, DPX-L, USA).

### NAFLD diagnosis via abdominal ultrasound

The sonographer used a 3–5 MHz probe to examine and evaluate the liver. The NAFLD diagnostic criteria based on ultrasound are the presence of signs of liver steatosis, such as bright liver echo patterns, increased echo beam attenuation, and loss of structural details in the liver [13].

### Collection of laboratory and baseline data

The basic information and laboratory examinations of the subjects were collected through the medical record system. Body mass index (BMI) is the weight (kg) divided by the standing height squared (m^2^) [14]. Laboratory data included total serum cholesterol (TC), TGs, high-density lipoprotein cholesterol (HDL-C), low-density lipoprotein cholesterol (LDL-C), creatinine, uric acid (UA), alanine transaminase (ALT), alanine aminotransferase (AST), glycosylated hemoglobin A1c (HbA1c), calcium, and fasting and postprandial blood sugar.

### Statistical analysis

Statistical analyses were performed with IBM SPSS (version 22.0, IBM SPSS Inc., Armonk, New York, US). Continuous standard variables are expressed as the mean ± standard deviation. Categorical variables are expressed in numbers (percentages) and were compared using the *χ*^2^ test. Student’s *t*-test was used to evaluate the difference in BMD between NAFLD and non-NAFLD groups. Linear regression analysis was used to evaluate the correlation between NAFLD and lumbar spine BMD. The average lumbar spine BMD was used as the dependent variable and selected variables based on the clinical background were independent variables for univariate regression analysis. The confounding factors with *p* < 0.1 in the univariate analysis were included in the multivariate analysis. To avoid multicollinearity, the variance inflation factor was evaluated before adjustment.

## Results

The baseline characteristics and laboratory data of the T2DM group and non-diabetes group are shown in Table 1.

**Table 1:** General characteristics of the study population

	Type 2 diabetes (*n* = 534)	Non-diabetes (*n* = 554)	*P*-value
Demographics			
Age (years)	69.5 ± 9.6	70.9 ± 9.8	0.177
Female	315(58.9)	343(61.9)	0.629
Height (cm)	158.3 ± 8.6	155.8 ± 9.1	0.001*
Body weight (kg)	62.2 ± 10.4	56.7 ± 11.9	0.001*
BMI (kg/cm2)	24.8 ± 0.4	23.4 ± 0.5	0.004*
Smoking history	137(25.6)	115(20.7)	0.835
Drinking history	114(21.3)	100(18.1)	0.706
Laboratory data			
HbA1c (%)	8.6 ± 2.3	5.6 ± 0.9	0.001*
T-chol (mmol/L)	4.75 ± 2.5	4.47 ± 1.1	0.075
Triglyceride (mmol/L)	2.2 ± 1.8	1.6 ± 2.5	0.165
HDL-C (mmol/L)	1.3 ± 0.3	1.4 ± 0.4	0.170
LDL-C (mmol/L)	2.6 ± 0.9	2.5 ± 1.1	0.326
ALT (U/l)	27 ± 26	23 ± 14	0.027
AST (U/l)	25 ± 19	27 ± 13	0.565
Creatinine (umol/L)	73 ± 56	64 ± 23	0.009*
Uric acid (umol/L)	292 ± 142	315 ± 108	0.025*
Calcium (mmol/L)	2.3 ± 0.1	2.2 ± 0.1	0.001*
BMD	0.91 ± 0.2	0.85 ± 0.2	0.025*
T-Score	− 1.1 ± 1.6	− 1.5 ± 1.76	0.001*
Z-Score	0.32 ± 1.3	0.25 ± 1.4	0.003*

The values are the mean ± SD, numbers in the brackets are percentages. *n* number of patients, *BMI* body mass index, *HDL* high-density lipoprotein cholesterol, *LDL* low-density lipoprotein cholesterol, *T-chol* total cholesterol, *AST* aspartate aminotransferase, *ALT* alanine aminotransferase, *HbA1c* glycosylated hemoglobin, *BMD* bone mineral density; *P < 0.05

There was no significant difference in age, sex, T-chol, triglyceride, LDL-C, AST, smoking or alcohol consumption history between the two groups (*P* > 0.05). The average body weight, BMI, TGs, ALT, UA, HbA1c, calcium, BMD, T-score and Z-score in type 2 diabetic group were higher than those in non-diabetic group (*P* < 0.05), the HDL-C, AST were lower than non-diabetic group (*P* < 0.05).

The relationships between lumbar spine BMD and NAFLD in different groups are shown in Tables 2, 3. Taking participants free from diabetes and NAFLD as the reference group, and using the lumbar BMD as the dependent variable, multivariate analysis found that in the T2DM group, regardless of whether or not with NAFLD, there was a difference in lumbar spine BMD between the patients and the reference group (Table 3). In the T2DM group, univariate analysis revealed an association of lumbar spine BMD with NAFLD (*P* < 0.05). After adjusting for confounding factors (BMI, sex, age, TGs, HDL-C, serum calcium, UA, ALT), NAFLD and lumbar spine BMD were still positively associated (*P* < 0.05). In the non-diabetes group, univariate analysis revealed that lumbar spine BMD was associated with NAFLD (*P* < 0.05). After adjusting for confounding factors (TGs, HDL-C, sex, age, ALT, UA, calcium), there was no correlation between lumbar spine BMD and NAFLD (*P* > 0.05) (Tables 2, 3).

**Table 2:** Univariate regression analysis: the effect of the study variables on lumbar spine BMD

	Type 2 diabetes				Non-diabetes			
	*β*	95% CI		*P* value	*β*	95% CI		*P*-value
Age (years)	− 0.035	− 0.05	− 0.02	0.001*	− 0.04	− 0.06	− 0.02	0.001*
Sex (male)	1.569	1.261	1.877	0.001*	1.553	1.153	1.954	0.001*
Height (cm)	0.081	0.059	0.103	0.001*	0.078	0.054	0.103	0.001*
Body weight (kg)	0.065	0.046	0.083	0.001*	0.081	0.063	0.098	0.001*
BMI (kg/cm2)	0.069	0.012	0.125	0.017*	0.16	0.097	0.224	0.001*
Smoking history	0.027	− 0.361	0.416	0.890	0.001	− 0.5	0.502	0.997
Drinking history	− 0.026	− 0.445	0.394	0.905	0.047	− 0.499	0.592	0.866
HbA1c (%)	0.017	− 0.059	0.094	0.653	0.26	− 0.453	0.972	0.472
T-chol (mmol/L)	− 0.011	− 0.46	0.124	0.872	− 0.051	− 0.248	0.147	0.615
Triglyceride (mmol/L)	0.26	− 0.02	0.163	0.126	0.186	− 0.041	0.413	0.109
HDL-C (mmol/L)	− 0.679	-1.167	− 0.19	0.007*	− 1.238	− 1.728	− 0.747	0.001*
LDL-C (mmol/L)	− 0.022	− 0.207	0.163	0.957	0.15	− 0.098	0.399	0.235
ALT (U/l)	− 0.699	-1.078	− 0.32	0.001*	− 1.238	− 1.897	− 0.578	0.001*
AST (U/l)	0.013	0.002	0.023	0.021*	0.021	0.007	0.036	0.004*
Creatinine (umol/L)	0.006	− 0.005	0.016	0.305	− 0.004	− 0.02	0.012	0.621
Uric acid (umol/L)	0.014	0.003	0.025	0.014*	0.009	0.000	0.018	0.056
Calcium (mmol/L)	0.014	0.000	0.004	0.044*	0.005	0.002	0.007	0.001*
NAFLD	0.699	0.32	1.078	0.001*	0.892	0.32	1.46	0.001*

*BMI* body mass index, *HDL* high-density lipoprotein cholesterol, *LDL* low-density lipoprotein cholesterol, *T-chol* total cholesterol, *AST* aspartate aminotransferase, *ALT* alanine aminotransferase, *HbA1c* glycosylated hemoglobin; *P < 0.05

**Table 3:** Multivariate linear analysis: the effect of the study variables on lumbar spine BMD

	*β*	*P*-value	95%Cl (%)	*R*^2^
All subjects				
T2DM with NAFLD	0.812	< 0.001	0.370–1.253	0.439
T2DM without NAFLD	0.567	0.001	0.220–0.915	
Non-diabetes with NAFLD	0.626	0.145	− 0.217–1.470	
Age	− 0.024	0.001	− 0.038– − 0.009	
Sex (male)	1.574	< 0.001	1.275–1.873	
BMI	0.948	< 0.001	0.593–1.304	
Subgroup				
T2DM				0.401
NAFLD	0.488	0.027	0.010–0.883	
BMI	0.085	0.004	0.04–0.155	
Age	− 0.03	0.005	− 0.047– − 0.004	
Sex (male)	1.366	0.001	1.038–1.956	
Non-diabetes				
BMI	0.107	0.031	0.040–0.238	0.498
Sex (male)	2.158	0.001	0.845–2.560	

All subjects groups, BMI, sex, age, TGs, HDL-C, calcium, UA, ALT, were adjusted. Participants free from diabetes and NAFLD as a reference group

Subgroup: T2DM group, BMI, sex, age, TGs, HDL-C, calcium, UA, ALT, were adjusted

Non-diabetes group, TGs, HDL-C, sex, age, ALT, calcium, UA were adjusted

BMI, body mass index; NAFLD, nonalcoholic fatty liver disease

## Discussion

This study assessed the correlation between nonalcoholic fatty liver disease and lumbar spine bone mineral density in type 2 diabetes and non-diabetes patients. It was found that although the lumbar bone mineral density in the type 2 diabetes group and non-type 2 diabetes group was higher than that in the non-nonalcoholic fatty liver group, only nonalcoholic fatty liver in the type 2 diabetes group had a statistically significant effect on lumbar bone mineral density after adjusting for confounding factors.

Previous studies have been conducted on the correlation between nonalcoholic fatty liver disease and bone mineral density, but the results are still controversial. A retrospective study found that nonalcoholic fatty liver disease harms male femoral neck bone mineral density, but positively affects lumbar spine bone mineral density in postmenopausal women [15]. Another study that used liver biopsy as a diagnostic method for nonalcoholic fatty liver disease found that the bone mineral density of the lumbar spine in the nonalcoholic fatty liver disease group was higher than that in the control group [16]. Nevertheless, there was no significant difference in femoral neck BMD between the two groups. These results show that nonalcoholic fatty liver disease does not reduce lumbar spine bone mineral density, or that nonalcoholic fatty liver disease increases lumbar spine bone mineral density, which is partly consistent with our findings.

We speculate that one reason for this difference in bone mineral density between the lumbar spine and the femoral neck may be related to different body fat distributions. Subcutaneous and visceral fat have different metabolic characteristics. They may have different effects on bone mineral density in different areas, and this correlation may vary with age and sex [17, 18]. Some studies have also observed that serum fetuin-A is elevated in nonalcoholic fatty liver disease patients. Studies have found that fetuin-A is elevated in bone tissue [19]. In addition to being an important inhibitor of ectopic calcification, fetuin-A can also affect the production of inflammatory mediators and participate in the regulation of bone metabolism [20–22], Nevertheless, research on fetuin-A is currently limited to in vitro experiments. In addition to metabolic factors, the effect of nonalcoholic fatty liver disease on the increase of lumbar bone mineral density may also be related to the structural characteristics of lumbar vertebrae. A study by Kirchengast et al. found that central or upper body fat distribution may influence hip bone mineral density [23]. Upper body fat can hinder bone loss in the spine. In addition, the lumbar spine has more metabolically active and hormone-sensitive trabecular bone than other sites [24].

Conversely, we also noticed that several cross-sectional studies have reported that nonalcoholic fatty liver disease is associated with a decrease in bone mineral density [25–27]. However, the subjects in these studies were mostly children and adolescents, and some were postmenopausal women. These findings indicated that the relationship between NAFLD and BMD is different among different people. At present, the mechanism underlying the low bone mineral density in adolescents and postmenopausal women with nonalcoholic fatty liver disease is not completely clear. This difference may also be related to differences in calcium, growth hormone, and insulin growth factors in different populations. In addition, circulating molecules may affect bone metabolism by affecting early childhood obesity [28].

Our study found that lumbar spine BMD increased in patients with T2DM. Most of the current studies support that the BMD of T2DM subjects is identical or higher than that of people without T2DM, which may be related to the higher obesity rate of T2DM patients. Obesity may lead to an increase in mechanical load and strain, thus increasing BMD [29, 30]. In addition, according to the current study, T2DM can affect many hormones that act on bone through endocrine pathways, thereby affecting bone metabolism and increasing bone formation [31].

Although there have been some studies on the relationship between nonalcoholic fatty liver disease and bone mineral density, few studies have examined the relationship between nonalcoholic fatty liver disease and bone mineral density in patients with type 2 diabetes. Our results showed that the presence of nonalcoholic fatty liver disease is an independent factor affecting the increase of bone mineral density in patients with type 2 diabetes. The pathophysiology of nonalcoholic fatty liver disease involves intestinal-derived microbial components, lipotoxicity and inflammation [32]. Excessive accumulation of adipose tissue in the liver increases the release of free fatty acids, which may be the main factor regulating insulin sensitivity [33]. Study found that nonalcoholic fatty liver may cause changes in several substances that affect bone mineral density, such as overproduction of osteopontin and reduced production of vitamin D and osteoprotegerin [34]. In addition, the use of hypoglycemic and lipid-lowering drugs in patients with diabetes and nonalcoholic fatty liver, as well as changes in diet, exercise and other living habits, may affect bone mineral density [35, 36]. These may be associated with higher lumbar bone mineral density found in the nonalcoholic fatty liver disease population, but the higher bone mineral density in the nonalcoholic fatty liver disease population does not prove a low risk of fracture in this population. At present, we have not found that nonalcoholic fatty liver disease people have relatively higher bone mineral density in bones other than lumbar vertebrae, so we think that there may be a more complex mechanism for this specific change of lumbar vertebrae.

This study has several limitations. First, in this study, we did not use liver biopsy to evaluate nonalcoholic fatty liver disease. Liver biopsy is the golden diagnosing standard for nonalcoholic fatty liver disease, but it is invasive and difficult to perform widely. Therefore, we chose abdominal ultrasound, which has been widely used in the clinic as a diagnostic method. Although ultrasound cannot accurately classify fatty liver, we believe that this method is a qualitative examination which is fully tested and reliable. Second, although the current research took into account many factors that are not included in previous research, it is still impossible to completely exclude all confounding factors, such as bone turnover biomarkers, vitamin D, and steroids. However, we believe that these factors would not significantly impact the results of this study. Third, our study is just a cross-sectional study, more prospective and mechanism-related studies are necessary to evaluate the relationship between type 2 diabetes, nonalcoholic fatty liver disease and bone mineral density.

## Conclusions

This study found that the strength of the association between nonalcoholic fatty liver disease and lumbar spine bone mineral density differs between individuals with and without diabetes. The impact of nonalcoholic fatty liver disease on bone needs to be evaluated in different clinical backgrounds. When clinical intervention is required, it is necessary to consider the different effects of different metabolic factors on patients. Larger prospective studies, especially those related to mechanisms, are needed in the future to better understand the relationship between nonalcoholic fatty liver disease, type 2 diabetes and bone mineral density and fracture risk.

## Data Availability

The supporting data can be acquired via the corresponding author.
